# Assessment of IAAF Racewalk Judges' Ability to Detect Legal and Non-legal Technique

**DOI:** 10.3389/fspor.2019.00009

**Published:** 2019-08-08

**Authors:** Brian Hanley, Catherine B. Tucker, Athanassios Bissas

**Affiliations:** Carnegie School of Sport, Leeds Beckett University, Leeds, United Kingdom

**Keywords:** athletics, biomechanics, force plate, testing, videography

## Abstract

IAAF Rule 230.2 states that racewalkers must have no visible (to the human eye) loss of contact with the ground and that their advancing leg must be straightened from first contact with the ground until the “vertical upright position.” The aims of this study were first to analyze racewalking judges' accuracy in assessing technique and, second, to measure flight times across a range of speeds to establish when athletes were likely to lose visible contact. Twenty racewalkers were recorded in a laboratory using a panning video camera (50 Hz), a high-speed camera (100 Hz), and three force plates (1,000 Hz). Eighty-three judges of different IAAF Levels (and none) viewed the panned videos online and indicated whether each athlete was racewalking legally. Flight times shorter than 0.033 s were detected by fewer than 12.5% of judges, and thus indicated non-visible loss of contact. Flight times between 0.040 and 0.045 s were usually detected by no more than three out of eight judges. Very long flight times (≥0.060 s) were detected by nearly all judges. The results also showed that what judges generally considered straightened knees (>177°) was close to a geometrically straight line. Within this inexact definition, IAAF World Championship-standard Level III judges were most accurate, being more likely to detect anatomically bent knees and less likely to indicate bent knees when they did not occur. For the second part, the men racewalked down a 45-m indoor track at 11, 12, 13, 14, and 15 km/h in a randomized order, whereas the women's trials were at 10, 11, 12, 13, and 14 km/h. Flight times, measured using an OptoJump Next photocell system (1,000 Hz), increased for the men from 0.015 s at 11 km/h to 0.040 s at 14 km/h and 0.044 s at 15 km/h, and for the women from 0.013 s at 10 km/h to 0.041 s at 13 km/h and 0.050 s at 14 km/h. For judging by the human eye, the threshold for avoiding visible loss of contact therefore occurred for most athletes at ~14 km/h for men and 13 km/h for women.

## Introduction

Racewalking is part of the athletics program at the Olympic Games, International Association of Athletics Federations (IAAF) World Championships, and all other major athletics events. Competitions are held over 20 km for men and women, with a 50 km event for women first added in 2017 to join the longstanding men's 50 km event. IAAF Rule 230.2 states that racewalking is a progression of steps with no visible (to the human eye) loss of contact with the ground and that the athlete's advancing leg must be straightened from first contact with the ground until the vertical upright position (IAAF, 2017). The “vertical upright position” was a term introduced by the IAAF more than 45 years ago, and is effectively the moment when the athlete's torso passes over the foot (IAAF, 1972). To ensure athletes are complying with Rule 230.2, qualified judges are positioned on the course to scrutinize the racewalking techniques used. If a judge observes an athlete exhibiting loss of contact or a bent knee, a red card is sent to the Chief Judge (IAAF, 2017). Three red cards from three different judges lead to disqualification (in international competition, the three judges must represent different nations), or in some events, the athlete suffers a time penalty (IAAF, 2017). Before issuing a red card, if a judge is not completely satisfied that a racewalker is complying with the rule, the athlete is shown a yellow paddle indicating the offense. Judges at major global championships, such as the Olympic Games, are selected from the IAAF's panel of Level III judges, who must perform well in written, verbal and video-based examinations. Judges at Area competitions (e.g., the European Championships) must be on at least the IAAF Level II panel, which also requires the passing of examinations. National competitions can be judged by individuals with IAAF Level I qualifications, which many nations incorporate into their own judge education programs.

It is important to note from IAAF Rule 230.2 that judging accuracy in identifying visible loss of contact and bent knees is equally important. As a result, paying attention to both aspects of the rule is crucial for athletes and coaches, as infringing either part can lead to disqualification: across all four 20 and 50 km races at the 2017 IAAF World Championships, seven athletes were disqualified for three red cards for loss of contact, six for three red cards for bent knees, and six for a mixture of contact and knee infringements (IAAF, 2019). Overall, 91 red cards were awarded for loss of contact (mostly in the 20 km races), and 57 for bent knees (mostly in the 50 km races). Although there have been many studies on the biomechanics of racewalking, very little research on judging itself has been conducted. Two studies from the early 1990s included experiments that found the judges assessed were typically unable to observe loss of contact when it lasted <0.04 s. However, there were very few judges assessed: the first study (Knicker and Loch, 1990) featured one international judge and two national judges, whereas the other used “a coach of long-standing international experience” (De Angelis and Menchinelli, 1992, p. 87). These studies were conducted before the current racewalking rule was introduced in 1995, and at the time meant that athletes did not have to have a straightened knee by first contact, only at the instant of the vertical upright position. New research is therefore warranted that reflects modern racewalking techniques and more up-to-date judging practices.

A key point to reiterate is that there are no current set definitions of legal loss of contact or bent knee angles, except that they are judged with the human eye by appointed judges. With the aid of video cameras, previous research has found flight times as high as 0.07 s (Knicker and Loch, 1990), although more modest mean values of ~0.03 s have been found in recent competition and laboratory studies (Hanley et al., 2014; Hanley and Bissas, 2017), below the 0.04 s threshold suggested by Knicker and Loch (1990) and De Angelis and Menchinelli (1992). Nevertheless, the mean flight time of 0.03 s contributed to a mean flight distance of 0.12 m per step, and longer flight distances were found to correlate with racewalking speed (Hanley and Bissas, 2016), showing that it is important for judges to be consistent with their decisions to avoid individual athletes gaining an advantage. With regard to knee angles, Cairns et al. (1986) defined knee straightness as 175° and angles beyond that as hyperextension. Similarly, Knicker and Loch (1990) defined straightness as between 175 and 185°, but these values should not apply to current racewalkers given those studies were conducted under the pre-1995 rule. In terms of the actual angles that occur, the results of a very recent laboratory study showed that the mean knee angle at first contact was 180° (Hanley et al., 2018), similar to that found in world-class competition (Hanley et al., 2014), in laboratory settings using high-speed cameras (Padulo et al., 2013), and using optoelectronic systems (Pavei and La Torre, 2016), but whether this matches judges' opinions has not been hitherto analyzed. Furthermore, it has not been established whether there are differences in accuracy between IAAF judging Levels, a potentially important factor in deciding the future direction of judge education.

Racewalking is a very technical event with its own unique gait pattern, determined by IAAF Rule 230.2 and the athlete's attempts to maximize speed and efficiency. Judging the racewalk event accurately is a difficult skill and subjective by its very nature, so it will therefore be very beneficial to conduct new research on this topic with a far greater number of judges than those of previous two studies (Knicker and Loch, 1990; De Angelis and Menchinelli, 1992), particularly now that an examination must be passed to join the judging pool, and because of the post-1995 change in definition of the governing rule. Recent advances in technology, including internet-based surveys and digital videography, mean that reaching a greater number of judges worldwide is now possible. Similarly, because it has been proposed that electronic chip insole technology should be incorporated into racewalking competitions from 2021 (IAAF, 2019) for the measurement of loss of contact, establishing how much flight time must occur for visible detection, and at what speeds these typically occur (or not), is timely. For coaches, knowing what other spatiotemporal values, such as those of step length and step frequency, are likely to occur at speeds when loss of contact is visible is useful in monitoring athletes' technique. Furthermore, unlike loss of contact, no attempt has been made previously to establish a “threshold” for bent knees, which would assist with coach, athlete and judge education. The aims of this study were first to analyze racewalking judges' accuracy in assessing technique and, second, to measure flight times across a range of speeds to establish, based on the judges' accuracy scores, at what speeds athletes were likely to begin to lose visible contact.

## Materials and Methods

### Research Approval

All human subjects were treated in accordance with established ethical standards. The protocol was approved by the Carnegie School of Sport Research Ethics Committee. All subjects gave written informed consent in accordance with the Declaration of Helsinki. The subjects were provided with Participant Information Sheets, and in accordance with the Carnegie School of Sport Research Ethics Committee's policies for use of human subjects in research, all subjects were informed of the benefits and possible risks associated with participation and informed of their right to withdraw at any time.

### Part 1: Data Collection and Analysis of Athletes for Judging Study

#### Participants

Twenty-six athletes from 12 nations participated in this part of the study. Of the 26, six athletes' videos were used for practice data in the following part of the study and excluded from all analyses. Of the remaining 20 athletes, nine were men (age: 23 ± 5 years, height: 1.76 ± 0.04 m, mass: 63.9 ± 5.1 kg, 20 km personal record (PR): 1:26:07 ± 6:37), and 11 were women (age: 26 ± 6 years, height: 1.68 ± 0.08 m, mass: 57.9 ± 10.3 kg, 20 km PR: 1:33:35 ± 6:19). One of the men was an U20 (junior) athlete who had not yet competed over 20 km. Ten of the 20 athletes had competed at the 2016 Olympic Games or 2017 IAAF World Championships.

#### Data Collection

Before testing, the athletes were given time to warm up and prepare, with practice trials taken to become accustomed to the laboratory setting. The participants racewalked along a 45-m indoor running track in the biomechanics laboratory on multiple occasions. The testing area was kept clear of equipment, and the floor cleared of any tape or markings. They were filmed using a Sony HXR-NX3 digital HD camcorder (50 Hz); the settings chosen were based on those recommended for upload to YouTube (1080/50p, progressive scanning, 1920 × 1080 px), which housed the videos (although they were accessed by judges in the second part of the study via online survey software and were not directly accessible on YouTube). The camcorder recordings were achieved using a panning technique, and the athletes racewalked both left-to-right and right-to-left. The video capture area was flooded with light from 26 overhead lights (~104 kW) that allowed very high-quality pictures to be obtained (the shutter speed selected was 1/1,750 s). The athletes were asked to racewalk at a variety of paces, completing at least six trials each, with the trial best suited to analysis chosen based on accuracy of contact with the force plates, quality of camera footage, and symmetry between left and right contact times (Tucker and Hanley, 2017).

High-speed video data were recorded simultaneously with the panning footage using a stationary camera (Fastec TS3, San Diego, CA). The resolution of the camera was 1280 × 1024 px, a 25 mm lens was used, the shutter speed was 1/2,000 s and the *f*-stop was 2.0. This camera was positioned ~10 m from and perpendicular to the plane of racewalking, and recorded movement over a distance of 5 m around the data capture area to allow for the calculation of knee angles. Four 3 m high reference poles were placed in the center of the camera's field of view in the center of the running track in the sagittal plane. The reference poles provided 12 reference points (up to a height of 2 m) that were later used for calibration (scaling) when calculating knee angles.

In the area where the high-speed camera was focused, the athletes racewalked across three adjacent force plates (9287BA, Kistler Instruments Ltd., Winterthur), from which any loss of contact time was measured (simultaneously with the cameras). These force plates (1,000 Hz) were 900 mm long and 600 mm wide (natural frequency ≈ 750 Hz (x-, y-), ≈520 Hz (z-); linearity < ±0.5% full scale output (FSO); cross talk < ±1.5%; hysteresis < 0.5% FSO) and placed in a customized housing in the center of the track. The force plates were covered with a synthetic athletic surface so that the force plate area was flush with the rest of the runway to preserve ecological validity (Bezodis et al., 2008), while still being separate from the surrounding surface.

#### Data Analysis

The video files were digitized manually (SIMI Motion 9.2.2, Munich) by a single experienced operator. Digitizing was started at least 15 frames before first contact (heel-strike) and completed at least 15 frames after toe-off to provide padding during filtering (Smith, 1989). Each video was first digitized frame by frame, and adjustments made as necessary using the points over frame method (Bahamonde and Stevens, 2006). The segment endpoints used to calculate the knee angle were the hip joint, knee joint, and ankle joint. A recursive second-order, low-pass Butterworth digital filter (zero phase-lag) was used to filter the raw knee angle data (Winter, 2005). The cut-off frequencies were calculated using residual analysis (Winter, 2005). The knee angle was calculated as the sagittal plane angle between the thigh and lower leg segments, and rounded to the nearest integer. Knee angles were considered to be 180° in the anatomical standing position, and angles beyond this as hyperextension. The knee angle has been presented in this study at specific events of the gait cycle as defined below:

- First contact—this was the first visible point during stance where the athlete's foot clearly contacts the ground (heel-strike).
- Midstance—this was a visually chosen position where the athlete's foot was directly below the hip, used to determine the “vertical upright position.”

The force data were smoothed using a recursive second-order, low-pass Butterworth filter (zero phase-lag) at 50 Hz (Hanley and Bissas, 2017). The mean and standard deviation (SD) of the noise occurring during the final 50 ms before ground contact (visual inspection) were calculated, and first contact was considered to begin when the vertical force magnitude was greater than the mean plus 3SD of the noise (Addison and Lieberman, 2015; Hanley and Tucker, 2019). The mean and 3SD of the noise during the first 50 ms after toe-off were used in a similar way to identify the end of contact and the beginning of flight (i.e., loss of contact). Flight time was defined as the time duration from toe-off of one foot to the first contact of the contralateral foot (Padulo et al., 2014).

### Part 2: Data Collection and Analysis of Judges

As previously described, 20 of the 26 athletes' videos were chosen to be part of the online judging test. Of the other six, five were chosen as “practice trials,” and one was chosen as a trial to allow participants to adjust their computer settings. Thirteen of the videos were of athletes racewalking from left-to-right, and seven from right-to-left. An online survey system, Qualtrics, was used to collect responses from judges.

The vid Tube to prevent access from unauthorized viewers. The following code was used to control how the videos played within Qualtrics (the YouTube playlist title beginning “xyz” in the example given below is fictitious):

- <iframe width=“960” height=“540” src=“https://www.youtube.com/embed/xyz123456?autoplay=1&amp;&loop=1&playlist=xyz123456;&rel=0&amp;showinfo=0&amp;modestbranding=1&amp;controls=0” width=“960” wmode=“transparent”>frameborder=“0” allowfullscreen>< /iframe> Please choose one of the following:

where:

- autoplay = 1—meant the video began automatically
- loop = 1—replayed the video on a continuous loop until the participant had answered the question and moved to the next video
- rel = 0—prevented related videos from appearing via YouTube at the end of each clip
- showinfo = 0—prevented the participant from viewing the video file details
- modestbranding = 1—restricted the amount of time the YouTube logo was visible on screen
- controls = 0—removed the option for participants to pause, play or slow the video

In addition, the code below created a transparent mask over each video so that participants could not click on it (which could have been used to pause it):

- <div style = “position: absolute;top:0px;left:0px;width: 1000px;height:570px;background-color: white;z-index:2;opacity:0.0;filter: alpha(opacity = 0)”> < /div>

Respondents were asked to watch the 20 videos in turn and to make a judging decision based on what they saw for each one. The symbols used in the survey for infringements reflected those used in competition: ~for loss of contact and > for bent knees.

We disseminated the survey directly from Qualtrics, via social media and via email. Email details for IAAF Level II and Level III judges were obtained from the IAAF, and access to the survey could be accessed by the email recipient only. Before beginning the survey, participants were provided with an online Participant Information Sheet and Informed Consent Form; all participants had to declare that they were at least 18 years old. Apart from responses to the actual racewalking videos, we used the Qualtrics system to request the following information from participants:

- Sex and age (one selection allowed): (male; female)/(18–29; 30–39; 40–49; 50–59; 60–69; 70+)
- IAAF Area of residence (one selection allowed): (Africa; Asia; Europe; North America; Oceania; South America)
- Involvement in racewalking (multiple selections allowed): (judge; administrator/governing body official; athlete; coach; supporter/fan; scientist/researcher; none)
- Judging qualifications (one selection allowed): (IAAF Level III; IAAF Level II; IAAF Level I; National qualification; none)
- Highest standard of competition judged at (one selection allowed): (World Championships/Olympic Games; Area competition; World age-group championships; Area age-group championships; International match/Regional championships; Local/regional championships; national championships; none).

The Qualtrics software also recorded data of total time spent on the survey per respondent and time spent per individual video. There was a total of 223 responses to the survey; of these, 121 were incomplete responses (in some cases, we were contacted directly by judges to say that they had begun the survey but then realized their YouTube settings were less than optimal, and began the survey again on another computer). Respondents were asked about their quality setting when using YouTube (as stated above, a non-assessed video was provided for respondents to alter their settings to the best quality available, with this setting automatically retained for the test videos). Because it would have led to unsuitably low video quality for judging, 17 respondents who stated that their resolution was lower than 480p were excluded from the final survey.

After excluding those who had inappropriate quality settings, 63 men and 22 women were included in the final sample; their Area and qualifications are shown in Table 1. As very few judges (*N* = 4) described themselves as being IAAF Level I, their responses include those who described themselves as having national qualifications. Responses from those who had no qualifications, where respondents could choose more than one option to describe their involvement in racewalking, showed that the largest subgroup were athletes (*N* = 15), with fewer describing themselves as either coaches (*N* = 8), racewalking supporters/fans (*N* = 7), scientists/researchers (*N* = 3), or administrators/governing body officials (*N* = 1). With regard to age groupings, five respondents were in the 18–29 category, 13 were in the 30–39 category, 19 were in the 40–49 category, 24 were in the 50–59 category, 17 were in the 60–69 category, and seven were 70 years old or older.

**Table 1:** Number of judges from each area at each level.

**Area**	**Level III**	**Level II**	**Level I**	**No qualification**	**Total**
Africa	0	0	3	1	4
Asia	1	1	3	0	5
Europe	10	8	15	14	47
North America	2	5	5	6	18
Oceania	0	1	6	1	8
South America	0	2	1	0	3
Total	13	17	33	22	85

### Part 3: Changes in Spatiotemporal Variables With Increased Speed

Twenty athletes participated in this part of the study, of whom 14 had also taken part in the judging video study. Eleven of these racewalkers were men (age: 26 ± 4 years, height: 1.77 ± 0.06 m, mass: 64.4 ± 4.7 kg, 20 km PR: 1:23:02 ± 2:28) and nine were women (age: 25 ± 4 years, height: 1.68 ± 0.09 m, mass: 57.5 ± 10.6 kg, 20 km PR: 1:32:23 ± 6:02). Fifteen of these athletes had competed at the 2016 Olympic Games or 2017 World Championships.

The men racewalked multiple times down the same 45-m indoor track as in the earlier part of the study at 11, 12, 13, 14, and 15 km/h in a randomized order, whereas the women's trials were at 10, 11, 12, 13, and 14 km/h. The time taken to cover the analyzed 5 m distance in the data capture area was measured using dual photocell Witty timing gates (Microgate, Bolzano, Italy), and had to be within 3% of the target time to be included for analysis. Flight times were measured for each trial at 1,000 Hz using five connected 1 m strips of an OptoJump Next system (Microgate, Bolzano, Italy). Results from the OptoJump Next system were extracted using specific settings (GaitIn_GaitOut) of 2_2 based on the number of light emitting diodes (LEDs) that formed the baseline and found to be optimal during a reliability study (Hanley and Tucker, 2019). The minimum threshold for flight time was set at 0.001 s (Hanley and Tucker, 2019). The variables extracted for this part of the study were step length, step frequency, contact time, and flight time.

#### Statistics

All statistical analyses were conducted using SPSS Statistics 24 (IBM SPSS, Inc., Chicago, IL). Results are presented as means and SD. Pearson's product moment correlation coefficient (*r*) and regression analysis were used to find associations between judges' response rates and flight times and knee angles; an alpha level of 5% was set. Effect sizes for correlations were either small (*r* = 0.10–0.29), moderate (0.30–0.49), large (0.50–0.69), very large (0.70–0.89), or extremely large (>0.90) (Hopkins et al., 2009). For the regression analysis, a component had to be statistically significant at the 0.05 level and account for at least 3% of the variance in detection rate score to be retained in the final model, whereby a polynomial regression analysis was employed to fit the data with an appropriate quadratic model. Coefficient of determination (*R*^2^) has been reported for the regressions (Field, 2009). Independent *t*-tests were conducted to compare values between men and women where appropriate, with adjustments made when Levene's test for equality of variances was <0.05 (Field, 2009). Effect sizes for differences between groups were calculated using Cohen's *d* (Cohen, 1988), rounded to two decimal places and considered to be either trivial (*d* ≤ 0.20), small (0.21–0.60), moderate (0.61–1.20), large (1.21–2.00), or very large (≥2.01) (Hopkins et al., 2009). On the occasions where Cohen's *d* was calculated, only those results where the effect sizes were moderate, large, or very large have been included. One-way repeated measures analysis of variance (ANOVA) was conducted at five speeds with repeated contrast tests conducted to identify changes between successive measurement speeds (Field, 2009). An alpha level of 5% was set for these tests with Greenhouse-Geisser correction used when Mauchly's test for sphericity was significant.

## Results

The results presented below first comprise the judging decisions made via Qualtrics with regard to both flight times and knee angles. Where appropriate, trendlines have been added to the data to show relationships. Extra lines have been used to show boundaries of judging to highlight meaningful values, in terms of what proportion of judges detected non-compliance with Rule 230.2. These boundaries are based on the proportion of judges required in the IAAF World Athletics Series and Olympic Games to award a red card to disqualify an athlete (three out of eight: 37.5%, red dashed lines), with the lower limit set at a proportion of less than one out of eight (<12.5%, green dashed lines) to indicate a very low likelihood of detection.

**Figure 1:**
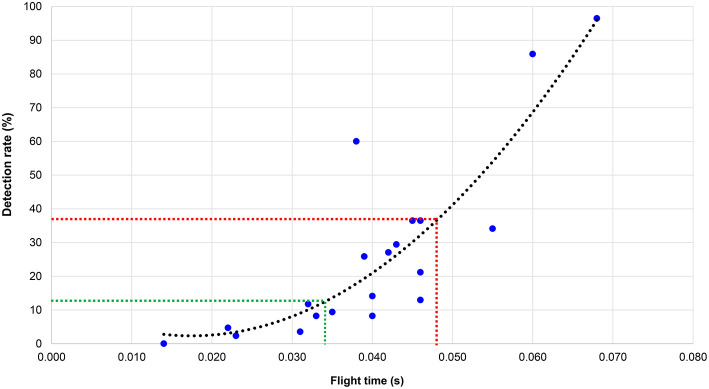
Detection rates for flight time for all 85 participants in the judging part of the study.

Figure 1 shows the detection rates for flight time for all 85 participants in the judging part of the study (each data point represents each analyzed athlete). The result from the regression analysis showed that the detection rate was: 13.0 – 1253 × flight time + 36315 × flight time^2^ [*F*_(2, 17)_ = 25.68, *p* < 0.001, *R*^2^ = 0.75]. The green dotted line shows that those flight times shorter than 0.033 s (six athletes) were detected by fewer than 12.5% of judges and are therefore likely to indicate non-visible loss of contact. Most flight times that were approximately between 0.040 and 0.045 s were in a detection zone where a proportion of one or two judges detected loss of contact, but not more than three out of eight. Very long flight times (≥0.060 s) were detected by more than 85% of judges.

**Figure 2:**
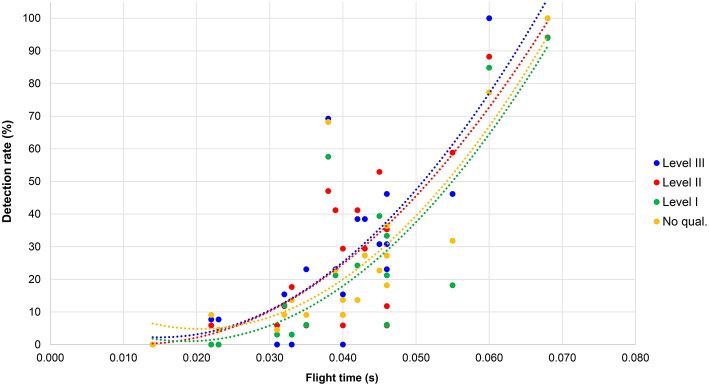
Detection rates for flight time for each group of judges by level.

Similar results from the regression analysis were found for each Level of judge (Figure 2): Level III—*F*_(2, 17)_ = 22.42, *p* < 0.001, *R*^2^ = 0.73; Level II—*F*_(2, 17)_ = 24.10, *p* < 0.001, *R*^2^ = 0.74; Level I—*F*_(2, 17)_ = 18.75, *p* < 0.001, *R*^2^ = 0.69; no qualifications—*F*_(2, 17)_ = 21.13, *p* < 0.001, *R*^2^ = 0.71. The most noticeable difference between the groups was that nearly 50% of Level III judges and 60% of Level II judges detected loss of contact in the athlete with ~0.055 s flight time, whereas for the Level I judges and those with no qualifications, it was between 18 and 32%.

**Figure 3:**
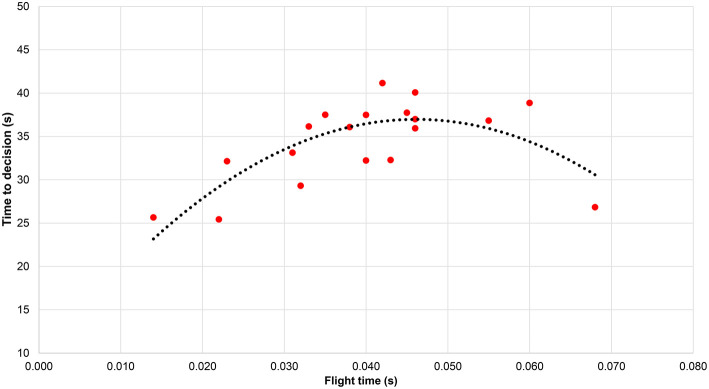
Mean time taken by all participants to make their decision for each individual (indicated by their flight time).

Figure 3 shows the mean time taken for all participants to make their decision for each video in terms of the individual flight times measured. The time to detection was: 8.5 + 1235 × flight time – 13382 × flight time^2^ [*F*_(2, 16)_ = 11.61, *p* = 0.001. *R*^2^ = 0.59], with its peak occurring at 0.046 s.

**Figure 4:**
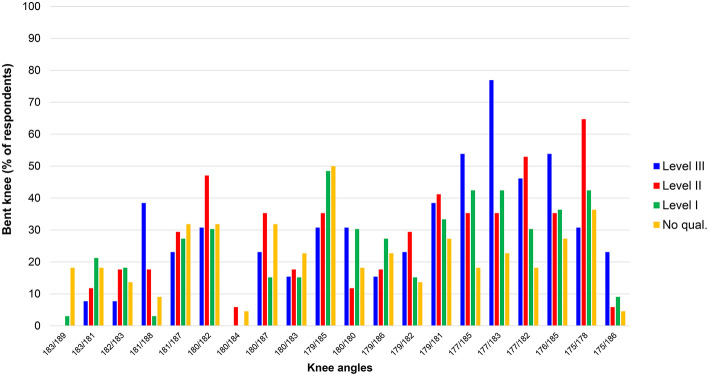
Detection rates for knee angles at first contact and midstance for all 85 participants in the judging part of the study.

**Figure 5:**
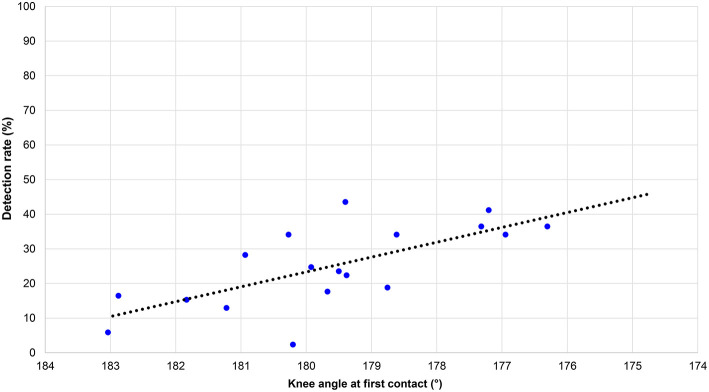
Detection rates for knee angles (first contact) with two outliers removed for all 85 participants in the judging part of the study.

Figure 4 shows the detection rate for knee angles for each Level of judge for each individual athlete; the horizontal axis shows the values for knee angle at both first contact and midstance. Nearly all athletes increased the knee angle after first contact, and all but one had hyperextended knees at midstance. In general, bent knees were most likely to be identified when the first contact angle was 179° or below. Because racewalking is a continuous movement, it is not possible to identify precisely whether the midstance angle affects perception of the knee's appearance at first contact. However, it was interesting to find that one athlete with a knee angle of 175° at first contact (on the far right of Figure 4) was not detected for bent knees, and it is possible that this was because this athlete's knee hyperextended to 186° by midstance (this athlete also had the shortest measured flight time). In addition, the detection rate for one other athlete (second from the right-hand-side of Figure 4) who had bent knees at both first contact (175°) and midstance (178°) was relatively low. In this athlete's case, the flight time was highest of all athletes and the detection rate for loss of contact was very high (100% amongst Level III judges). These two athletes' results can therefore be considered outliers regarding detection of bent knees, and are removed from Figure 5, which shows the detection rates for all judges for knee angles at first contact (*r* = 0.70, *p* = 0.001). So that they appear in a similar format to the flight time data (with more “legal” values found on the left of the scale), the knee angles are presented with hyperextended values on the left and decrease along the horizontal axis, and the correlation values reported below are represented as positive values.

**Figure 6:**
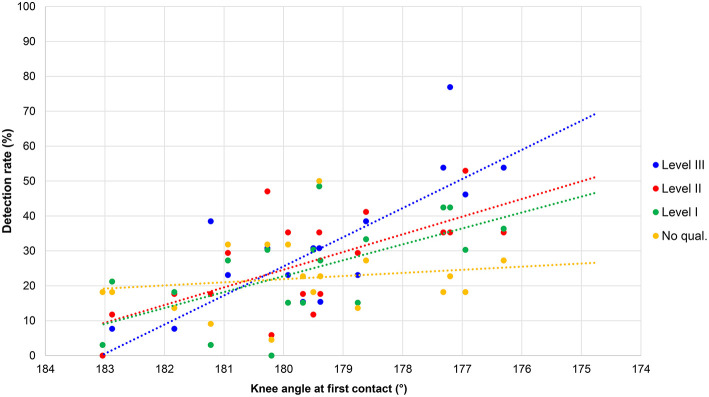
Detection rates for knee angles (first contact) for each group of judges by level.

Level III judges were the best at making correct calls for knee straightness (*r* = 0.79, *p* < 0.001) (Figure 6), in that they were more accurate at detecting bent knees when they did occur, and more accurate at correctly identifying legal knee straightness (i.e., lower bent knee detection rates for knees when they were straight). Level II and Level I judges were roughly equal in their ability to make correct knee decisions (*r* = 0.67, *p* = 0.002 and *r* = 0.62, *p* = 0.006, respectively). However, those respondents without any judging qualifications were poor at this task (*r* = 0.17, *p* = 0.507), in that they did not differentiate between those with bent knees and those with legal knees (up to 183° extension; i.e., hyperextension). There was no correlation between knee angle and the time taken to make a decision.

**Figure 7:**
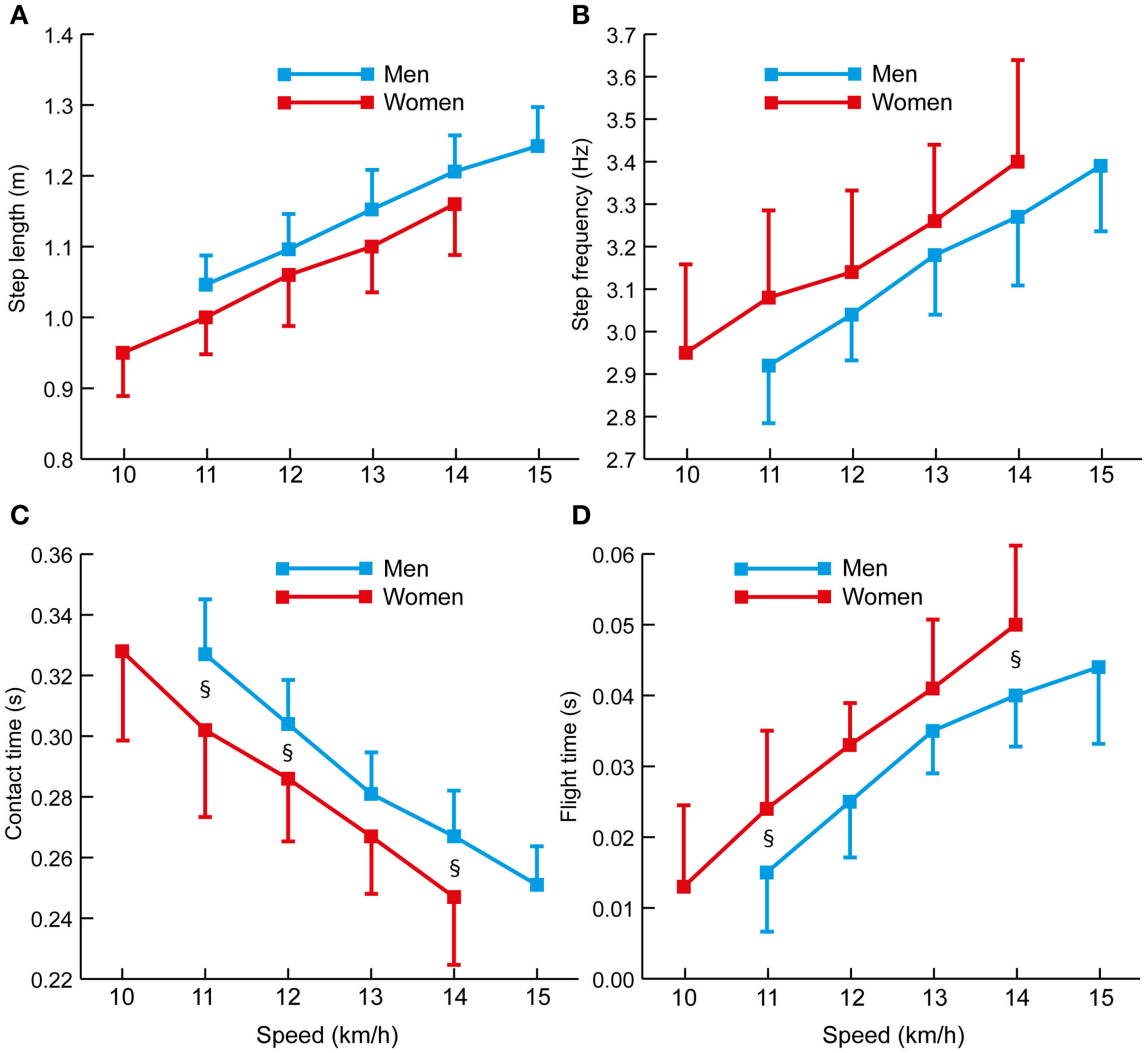
Changes in spatiotemporal variables with increased speed in men and women racewalkers: **(A)** step length, **(B)** step frequency, **(C)** contact time, and **(D)** flight time. Results are shown as means, with SD indicated as plus or minus for either group for clarity. Differences between men and women (*p* < 0.05, *d* ≥ 0.61) are indicated with the § symbol.

In the separate part of the study on the effects of increased racewalking speeds, flight time increased between each successive speed amongst the men (*p* < 0.05, *d* ≥ 0.61) (Figure 7); however, there was no difference between 14 and 15 km/h. All other variables increased between successive speeds for men (*p* < 0.05, *d* ≥ 0.61). Amongst the women, all variables increased between successive speeds (*p* < 0.05, *d* ≥ 0.61) except for step frequency, which did not change between 11 and 12 km/h.

## Discussion

The aims of this study were first to analyze racewalking judges' accuracy in assessing technique and, second, to measure flight times across a range of speeds to establish when athletes were likely to lose visible contact. The study confirmed that, as occurs in elite-standard competition, each athlete had some flight time, replicating what has been shown in biomechanical research in competition (Hanley et al., 2014) and in laboratory testing (Hanley and Bissas, 2017). One key finding was that loss of contact was detected at similar rates for all groups, regardless of judge qualification status. This indicates that the inability to detect loss of contact below ~0.045 s is normal and representative of the human visual system. This finding concurs with the previous findings of Knicker and Loch (1990) and De Angelis and Menchinelli (1992) and emphasizes that the human eye cannot detect very short flight times. It also suggests that 0.040–0.045 s is an appropriate threshold to adopt as “visible loss of contact” in the absence of judges when coaches are testing athletes using electronic aids. By contrast, very low detection rates occurred when flight time lasted <0.033 s. Judges also took longer to make a decision when flight times were ~0.045 s and showed the need for judges to observe athletes on several occasions to make a decisive judgement, as is normal during a race. It is worth bearing in mind that the presented results were collecting using an experimental design in laboratory-based conditions. Although the results showed that most athletes (65%) had flight times between 0.03 and 0.05 s, indicating that these are the ranges typically adopted by well-trained racewalkers, the threshold of ~0.04 s for visible loss of contact that we (and previous research) found might not apply in race conditions where, for example, there are frequently large groups of athletes and judges' views are obstructed. Additionally, racing conditions differ from the laboratory because of weather conditions (e.g., bright sunlight, heavy rain), surface levelness, and racewalkers' motions that can deviate from a straight line. For training purposes, or for assessing judges in competition, technology could help, for example, by using a video camera to record athletes as they pass judges' positions (Hanley et al., 2018).

Any coaches who use technology should consider that modern racewalkers typically have flight times across a range of competitive speeds (from 0.01 to 0.05 s, approximately). Laboratory and field-based technology that is currently used to measure flight times includes force plates, high-speed video cameras, infrared optoelectronic systems, LED-based hardware, pressure insoles, and inertial sensors (Di Gironimo et al., 2017). Those systems that are external to the athlete (e.g., video cameras) have the advantage of being controlled by the coach or scientist without interfering with the athlete's movements (e.g., that inertial sensors might do). Technology for adjudicating on critical incidents has become more widespread in high-performance sport (e.g., cricket, football, and tennis). However, it should be noted that any introduction of technology to judge loss of contact from 2021 will require changes in racewalking techniques, which will have implications for athletes and their coaches, especially if the technology's threshold is set much differently from what the human eye is capable of. Care should be taken amongst coaches that training for the event does not become focused on any technology used and how to work within it, at the expense of the biomechanical or physiological optimization of movement.

The results showed that, as expected, fewer athletes were considered to have bent knees when the knee angle was larger (angles above 180° are hyperextended and abnormal in normal walking and running). Fewer than 20% of respondents considered knee angles above 181° to be bent, with a boundary of 177° as the lower limit for bent knee detection (i.e., more than 37.5% of judges). This effectively shows that knee straightness is considered to occur at angles above 177°, which is very close to the geometrical definition of a straight line (180°). In contrast to the detection rates for loss of contact, however, there was a clear difference between Levels of judge with regard to detection of knee infringements. There is no precise definition of what constitutes “straightened” or “bent” knees within IAAF Rule 230.2, and thus it is not a simple case of measuring a knee angle and comparing it to a standard value. Nonetheless, within this inexact definition, Level III judges were the best at judging knees, in that they were more likely to detect anatomically bent knees and less likely to indicate bent knees when they did not occur. Level II judges were also good at making correct decisions regarding knees, with little difference from Level I judges. By contrast, those who had no judging qualifications made both Type I errors (detecting bent knees when they did not exist) and Type II errors (not detecting bent knees when they did exist). The role of the judge is vital to racewalk competitions and their abilities to detect infringements can be improved, as shown by the better detection rates of higher qualified judges. Whereas, the detection of short flight times is more difficult when using the human eye because of its natural inability to detect very brief stimuli, judging knee infringements has been shown to be a skill more readily learned and a key area for future judge education. In this regard, judging racewalking is a serial visual search task, i.e., the judge has to scan the racewalkers to detect infringements, which is a task that is made more difficult when athletes racewalk in large groups (e.g., in big competitive fields like the World Race Walking Team Championships). Future scientific studies on racewalk judging could consider using eye-scanning technology to identify what the best judges are looking at when making correct decisions, with the results accrued used in future judge education. This will be especially important given the better detection rates of Level III judges in terms of bent knees, and some instances of more accurate detection of flight times of certain athletes by Level II and Level III judges.

For the athletes analyzed in the part of the study that required them to adopt a range of speeds, it was clear and unsurprising that flight time increased as racewalking speed increased: for the men from 0.015 s at 11 km/h to 0.040 s at 14 km/h and 0.044 s at 15 km/h, and for the women from 0.013 s at 10 km/h to 0.041 s at 13 km/h and 0.050 s at 14 km/h. It was noticeable that women had longer flight times than men at 14 km/h, even though there were no differences in step length or step frequency, and women might therefore be at a greater risk of visible loss of contact at racing speeds than men. This does not mean that flight time cannot be reduced by an athlete, and indeed the men did not have longer flight times at 15 km/h than at 14 km/h, suggesting that better technique can be achieved at faster speeds that the athlete has become accustomed to in high-intensity training and competition. At the relatively “safe” racewalking speed of 14 km/h, the men had step lengths and step frequencies of ~1.20 m and 3.25 Hz, respectively; similarly, for women at 13 km/h, their step lengths and step frequencies were ~1.15 m and 3.25 Hz. Athletes who wish to race faster, with spatiotemporal values greater than these, must ensure that they simultaneously develop technique to avoid increasing flight time. Additionally, the values give an indication as to the effect of any particular threshold for loss of contact (using appropriate technology) on racewalking speed to athletes, coaches and administrators. However, the results do not indicate that it is not possible to achieve faster times with no loss of contact whatsoever (the athletes were not instructed to attempt this), and therefore a different approach to technique could allow for less or no loss of contact.

Overall, the results showed that responses were quicker when the racewalker had either a very short flight time (invisible to the human eye) or a very long one. It took longest for judges to decide whether loss of contact occurred when the flight times were ~0.045 s, which as described above was about the threshold for clear loss of contact to the human eye. In practice, decision times of 40 s meant watching the video three or four times, whereas the quicker decisions (<30 s) required two or three views only. Judges therefore do need to observe athletes on more than one occasion during a competition before deciding to write a red card, and supports the current practice of showing a yellow paddle before issuing a red card (apart from in exceptional circumstances), as well as the practice of viewing each video three times during IAAF judge examinations. Coaches should also remind their athletes to adjust their technique when a paddle is shown, or when a warning is indicated on the board, as many ignore these valuable sources of feedback (Alves et al., 2018). Two outlying, or at least unusual, results were found in the detection of bent knees. One of these athletes was probably judged to have been legal given the knee angle increased to a hyperextended position of 186° by midstance, and the other athlete had a very long flight time (0.068 s). It is probable that, in this latter example, the judges detected flight very readily and did not concern themselves about knee angles. In actual competition, such a decision would be very natural given that judges can award only one red card to an athlete, and making such an easy decision is of benefit when judges have to observe many athletes in any particular race.

The format of this study was similar to how IAAF judging examinations (video component) are conducted: prospective members of the IAAF judging panels watch a series of 20 videos and are asked to indicate whether each identified athlete is racewalking legally. The main differences were that, in this study, judges could view the videos repeatedly, rather than a set number of times; the athletes racewalked in a laboratory, rather than in competition; and the athletes were filmed individually, rather than in a group (the IAAF judging videos comprise a mixture of individual and group shots). These limitations of the videoing process in this study were necessary to ensure that valid and reliable data were obtained from the force plates and high-speed camera. As stated above, the nuances of competition, such as the race surface, presence of large groups, and weather conditions, can all affect a judge's perception of the racewalkers' movements. In addition, judging in competitive situations has a real effect on the athlete (possible disqualification) and judges need to be completely sure that the athlete has infringed; in our study, there was no such pressure to be cautious and judges might have been more disposed to indicate perceived rule infringements. Because of these differences, research that compares these video-based decisions with “live” decisions made during competition would be invaluable, not just to evaluate any introduction of technology but also to evaluate the video-based assessments currently adopted in IAAF judge evaluation and selection. Accordingly, follow-up studies on racewalk judging could involve measurements taken with appropriate technology during competitions across different standards. Despite our best efforts to contact all appropriate participants, it was nonetheless difficult to recruit a large sample of judges (partially because the actual number of judges in the IAAF Level II and Level III panels is restricted to only the best judges) and future research should try to ensure as many judges as possible are involved.

## Conclusions

This was the first study to analyze the detection rates of a large number of qualified IAAF racewalking judges from around the world. On average, racewalkers had flight times across a range of speeds, and all Levels of judge had higher detection rates when flight time exceeded 0.045 s, and lower ones below 0.033 s. With electronic chip insole technology being currently developed, these values give a guide as to the realistic visual threshold of judges that could be replicated with technology; for coaches, the important finding was that these thresholds corresponded to racewalking speeds of ~14 km/h for men and 13 km/h for women. One of the most important findings was that there was little difference between Levels of judge in terms of detecting flight time, as the human visual system is unable to detect very brief loss of contact, regardless of judge qualification. In some instances of moderate loss of contact in an individual athlete, Level II and Level III judges had higher rates of detection, and indicates that for certain techniques, a degree of learned skill is involved. Study participants with no judging qualifications were as likely to decide that legal racewalkers were breaking the knee part of Rule 230.2 as much as those who had much more bent knees, whereas Level III judges were the best in this regard. Given it is proposed that insole technology is adopted to measure loss of contact, it is vital that world and national governing bodies strengthen the education of judges with regard to the identification of bent knees in particular, which were the sole reason for the disqualification of six athletes from the 2017 IAAF World Championships.

## Data Availability

The datasets generated for this study are available on request to the corresponding author.

## Ethics Statement

All human subjects were treated in accordance with established ethical standards. The protocol was approved by the Carnegie School of Sport Research Ethics Committee. All subjects gave written informed consent in accordance with the Declaration of Helsinki. The subjects were provided with Participant Information Sheets, and in accordance with the Carnegie School of Sport Research Ethics Committee's policies for use of human subjects in research, all subjects were informed of the benefits and possible risks associated with participation and informed of their right to withdraw at any time.

## Author Contributions

BH, CT, and AB conceptualized and designed the study and wrote the manuscript. BH and CT conducted the data collection and analyses and created figures and tables. All authors read and approved the final manuscript.

### Conflict of Interest Statement

The authors declare that the research was conducted in the absence of any commercial or financial relationships that could be construed as a potential conflict of interest. The reviewer JG-E declared a past co-authorship with one of the authors BH to the handling editor.
